# Development of Sustainable Extraction Processes for the Recovery of Antioxidants from *Punica granatum* L. Peel

**DOI:** 10.3390/foods15203764

**Published:** 2026-10-21

**Authors:** Simona Sagona, Francesco Margheriti, Matteo Banti, Fabio Di Ricco, Lorenzo Raffellini, Clementina Manera, Giuseppe Saccomanni, Filippo Fratini, Marinella De Leo, Simona Rapposelli

**Affiliations:** 1 Department of Veterinary Sciences, University of Pisa, Viale delle Piagge 2, 56124 Pisa, Italy; simona.sagona@unipi.it (S.S.); filippo.fratini@unipi.it (F.F.); 2 BioStructures Lab, Istituto Italiano di Tecnologia (IIT@NEST), 56127 Pisa, Italy; francesco.margheriti1994@gmail.com; 3 Laboratorio NEST, Scuola Normale Superiore, Piazza San Silvestro 12, 56127 Pisa, Italy; 4 Department of Pharmacy, University of Pisa, Via Bonanno 6, 56100 Pisa, Italy; matteo.banti@phd.unipi.it (M.B.); fabio.diricco@phd.unipi.it (F.D.R.); lorenzo.raffellini@phd.unipi.it (L.R.); clementina.manera@unipi.it (C.M.); giuseppe.saccomanni@unipi.it (G.S.); marinella.deleo@unipi.it (M.D.L.); 5 Center for Instrument Sharing, University of Pisa (CISUP), Lungarno Pacinotti 43, 56126 Pisa, Italy; 6 Interdepartmental Research Center Nutrafood, Nutraceuticals and Food for Health, University of Pisa, 56124 Pisa, Italy

**Keywords:** circular economy, antioxidants, *Punica granatum* peel, fermentation, antibacterial activity

## Abstract

Background: Pomegranate peel, a major by-product of the food industry, represents an underexploited source of valuable bioactive compounds. In the framework of a circular economy approach, this study aimed to develop a sustainable and solvent-free extraction strategy based on *Saccharomyces cerevisiae* fermentation for the recovery of antioxidant and antimicrobial phytochemicals from pomegranate peel waste. Methods: Pomegranate peel was subjected to fermentation for 18, 24, 48, and 72 h, and the resulting extracts (YE_18h, YE_24h, YE_48h, and YE_72h), together with a conventionally obtained chemical extract (CE), were evaluated for their antioxidant activity. Based on its superior antioxidant activity, the extract obtained after 48 h of fermentation (YE_48h) was selected for ellagic acid quantification by HPLC and characterization by UHPLC-HRMS (Ultra-High-Performance Liquid Chromatography coupled to High-Resolution Mass Spectrometry). Antibacterial activity of YE_48h, in comparison with CE, was assessed against six Gram-positive and Gram-negative bacterial strains. Results: Among the fermented samples, the extract obtained after 48 h of fermentation (YE_48h) exhibited the highest antioxidant activity, showing an IC_50_ value lower than that of a conventional chemical extract. Quantitative HPLC analysis revealed a remarkable enhancement in ellagic acid content, with YE_48h containing approximately 18-fold higher levels of ellagic acid than the conventional chemical extraction. UHPLC-HRMS fingerprinting further demonstrated the presence of additional bioactive phenolic compounds, including punicalagin isomers I and II and pedunculagin isomer I, which are known for their antioxidant and antimicrobial properties. The antibacterial assay demonstrated that fermentation modulated the antimicrobial profile of the extracts in a strain-dependent manner. Conclusions: *S. cerevisiae*-mediated fermentation is an efficient and environmentally friendly approach for the valorization of pomegranate peel by-products, enhancing the release of bioactive compounds while generating extracts with promising antioxidant and selective antibacterial properties.

## 1. Introduction

In recent years, the concept of a circular economy has gained significant attention as a sustainable alternative to the traditional linear economic model. The circular economy aims to enhance resource efficiency by minimizing waste and repurposing discarded materials, transforming them into valuable resources [1]. Against this background, a branch of science focuses on how to reduce and valorize food waste by using and recycling discarded food parts, demonstrating that these materials contain a high number of beneficial properties, including antioxidant and antimicrobial activities useful for producing nutraceuticals and health-related products [2]. Many studies have shown that these by-products, often discarded as waste, are in fact rich sources of bioactive compounds with remarkable antioxidant and nutraceutical properties, making them promising candidates for the development of health-promoting products [2]. Among the various food by-products, several studies have focused on the characterization of pomegranate (*Punica granatum* L., Lythraceae) waste [3,4]. The pomegranate fruit consists of three main parts: the arils, the seeds and the peel, which account for approximately 40%, 10% and 50% of the total fruit weight, respectively [5]. Pomegranate seeds vary in color from white to purple, and are embedded in a white, spongy, astringent membrane surrounded by a thick reddish skin called the mesocarp [6]. The arils contain about 85% water, 10% sugars, mainly fructose and glucose, and 1.5% pectin. The arils are also a rich source of bioactive compounds such as phenols, flavonoids, and anthocyanins [7]. Pomegranate seeds are a source of fatty acids, including punicic acid, an omega-5 fatty acid with promising health benefits [8,9]. The peel, or pericarp, contains various minerals, mainly potassium, calcium, phosphorus, magnesium and sodium, as well as complex polysaccharides [10]. Of particular interest is the peel’s high content of polyphenolic compounds, including flavonoids, proanthocyanidins, and ellagitannins, especially punicalagins, which are widely recognized for their antioxidant and anti-inflammatory properties and their crucial role in health promotion and disease prevention [11,12]. Ellagitannins are formed from gallotannins through intermolecular carbon-carbon coupling between at least two galloyl units, producing hexahydroxydiphenoyl acid, which spontaneously lactonizes to ellagic acid in aqueous solution [12]. Ellagic acid is released from ellagitannins in the gastrointestinal tract and is further metabolized by the human colonic microbiota into bioavailable compounds known as urolithins [13]. Several studies highlight the numerous health-promoting properties of ellagic acid and its metabolites, including marked anti-inflammatory, antiproliferative, antioxidant and anti-aging activity [14].

Ellagic acid is one of the main naturally occurring antioxidant agents and its intrinsic antioxidant ability is attributed to its free radical-scavenging activity, which has been suggested to be like that of essential vitamins [15,16]. This property extends not only to ellagic acid itself but also to its metabolites, which likewise exhibit strong antioxidant activity and high efficacy in eliminating free radicals [16]. In addition to its antioxidant potential, ellagic acid and its derivatives have been extensively studied for their antimicrobial properties. Several studies have confirmed the efficacy of methanolic, ethanolic and aqueous extracts containing ellagic acid against *Staphylococcus aureus*, *Proteus vulgaris*, *Escherichia coli*, *Klebsiella pneumoniae*, *Bacillus subtilis*, *Pseudomonas aeruginosa*, *Salmonella typhi* and *Candida albicans* [17,18,19,20]. The antibacterial activity of these compounds has been attributed to their amphipathic nature, which enables them to interact both with the hydrophilic molecules on the bacterial membrane and surface and with the hydrophobic inner regions of the membrane [17]. This study aims to explore the bioactive potential of a food industry by-product: pomegranate peel extracts obtained through a sustainable extraction process based on *Saccharomyces cerevisiae*, which should enhance the yield of bioactive compounds by promoting their release from the structural polysaccharide network of the peel. This effect is mediated by the production of hydrolytic and metabolic enzymes, including cellulases, pectinases and β-glucosidases [21], which facilitate the disruption of the cell wall matrix and increase the extraction efficiency. In this study, we characterized yeast-derived extracts and compared them with extracts obtained through strong acid extraction (sulfuric acid) and organic solvent (hydroalcoholic acetic acid solution). The extracts, namely yeast extract (YE) and chemical extract (CE), respectively, were evaluated for their ellagic acid content, antioxidant capacity, and antibacterial activity. The findings contribute to the growing body of knowledge supporting the circular economy by providing innovative strategies for waste valorization and further highlighting the considerable potential of food by-products as valuable resources for the development of health-promoting and antibacterial products. Although previous studies have characterized pomegranate peel extracts and explored microbial fermentation as a strategy to improve the availability and bioactivity of phenolic compounds, these approaches have rarely been combined with an integrated analytical evaluation of the resulting products. In most cases, investigations have relied on single or partially overlapping measurements, so that the relationship between the chemical composition of the extracts and their biological activities remains incompletely defined. To address this gap, the present work combines a sustainable extraction of pomegranate peel with a comprehensive analytical and functional characterization. In this integrated framework, each analytical level contributes a distinct piece of information: ellagic acid quantification demonstrates the release of free ellagic acid achieved by the fermentation process, by direct comparison with conventional chemical extraction; DPPH antioxidant assay quantifies the resulting enhancement of radical-scavenging capacity; UHPLC-HRMS profiling verifies whether additional metabolites are simultaneously enriched during fermentation, and antibacterial testing against selected Gram-positive and Gram-negative strains translates these compositional changes into functional evidence. Since antibacterial effects rarely depend on a single molecule, the parallel use of targeted quantification and untargeted profiling enables a discussion of possible synergistic interactions among bioactive compounds, rather than attributing the observed activity solely to ellagic acid. Overall, this combined evaluation provides a comprehensive assessment of both the chemical composition and the functional properties of the extracts, allows the correlation of specific phytochemicals with biological activities, and helps elucidate the biochemical transformations occurring during fermentation.

## 2. Materials and Methods

### 2.1. Chemicals and Reagents

Glucose (purity ≥ 99.5%), absolute ethanol (≥99.8%, GC), sulfuric acid (95.0–98.0%), methanol (gradient grade, ≥99.9%), HPLC/UHPLC Plus water, dimethyl sulfoxide (DMSO, Molecular Biology grade), formic acid (reagent grade, ≥95%) and acetonitrile (gradient grade, ≥99.9%) were purchased from Merck (Darmstadt, Germany). Glacial acetic acid (99.0%) and ellagic acid (97%), used as the reference standard, were obtained from Fluorochem (Hadfield, Glossop, United Kingdom). 2,2-Diphenyl-1-picrylhydrazyl (DPPH, cod. D9132), a stable free radical supplied at quality level 100 without a declared purity percentage, was purchased from Merck (Darmstadt, Germany). The UHPLC-grade methanol-water solution used for sample preparation was obtained from Deltek (Pozzuoli, Italy). Brain Heart Infusion (BHI) broth was purchased from Liofilchem S.r.l. (Roseto degli Abruzzi, Teramo, Italy). *Saccharomyces cerevisiae* used for fermentation was a commercial active dry brewer’s yeast (batch AT1008, Erbamea, Perugia, Italy). All other solvents and chemicals were of analytical grade and used as received.

### 2.2. Sampling Pomegranate Fruit Peel

Pomegranate peels (*Punica granatum* L.) were collected in September 2024 in the Lucca province (Tuscany, Italy) and stored at −20 °C until analysis. Samples were thawed and placed in a stove at 40 °C until completely dry. They were then homogenized and ground with a ceramic mortar to obtain a fine powder.

### 2.3. Preparation of Chemical Extract

Chemical extraction was performed according to the protocol of Lu & Yuan [22]. Briefly, 16.73 g of powder was suspended in 100 mL of extraction solution (distilled water: absolute ethanol: glacial acetic acid, 10:10:1) and stirred at 80 °C for 2 h. The mixture was then centrifuged at 4000 rpm for 30 min (MPW-352 centrifuge, MPW Med. Instruments, Warsaw, Poland) and the supernatant was collected. The obtained extracts, dark brown in color, were combined and concentrated by rotavapor (Rotavapor R-114, Büchi, Flawil, Switzerland). Distilled water was added to the extract (7 g) to a final volume of 100 mL and subsequently the mixture was hydrolyzed with 4% (*v*/*v*) sulfuric acid at 105 °C for 4 h. The resulting product was filtered through a porous filter funnel (40–100 µm), and the residue was washed twice with distilled water, crystallized from methanol and finally dried in a stove at 40 °C for 2 days, yielding 1.623 g of final chemical extract (CE, yield: 9.70%).

### 2.4. Preparation of Yeast Extracts

Extraction by fermentation process of brewer’s yeast was in accordance with the protocol of Wang and colleagues [23,24]. Briefly, 0.40 g of *Saccharomyces cerevisiae* (batch AT1008, Erbamea, Perugia, Italy) was mixed with 0.70 g of glucose and 50 mL of distilled water. The inoculum concentration was approximatively 1.05*10^10^ CFU/mL, determined using the serial dilution method and plating on Yeast Extract Peptone Dextrose (YPD) selective medium (Thermo Fisher Scientific S.p.A., Segrate, Milan, Italy). A total of 40.0 g of dried pomegranate powder was suspended in 200 mL distilled water, and the entire yeast solution (50 mL) was added, giving a final fermentation volume of 250 mL. Fermentation took place in a sterile environment in glass containers at 30 °C for 72 h. Within this interval, 4 aliquots of 10 mL were collected at times 18, 24, 48, and 72 h. At each collection time, the pH of the fermenting mixture was measured and was required to remain above 5 [24,25]. All samples, regardless of collection time, were stored at −20 °C to stop the fermentation process. For analysis, the samples were thawed and centrifuged at 6000 rpm for 30 min (MPW-352 centrifuge, MPW Med. Instruments, Warsaw, Poland). The supernatant (220 mL) was then filtered through a filter funnel (40–100 µm), and the fermented aqueous suspension was evaporated to dryness by rotavapor (Rotavapor R-114, Büchi, Flawil, Switzerland), yielding 4.0 g of final YE (yield: 10%).

### 2.5. DPPH Radical Scavenging Assay

The CE and YE extracts (3 mg) and the ellagic acid were mixed with DMSO (6 mg/mL), and the radical scavenging activity (RSA) of the samples was evaluated using the DPPH (2,2-di(4-tert-octylphenyl) -1-picrylhydrazyl) free radical assay [26]. Briefly, 20 µL of different concentrations of the samples (from 0.6 mg/mL to 0.029 × 10^−3^ mg/mL) were mixed with 180 µL of DPPH solution (7 mM) in a 96-well plate. The obtained mixture was incubated for 45 min at room temperature in darkness. The neutralization of the DPPH radical leads to a decrease in absorbance, monitored against a blank at 510 nm using an EnSpire UV-Vis spectrophotometer (PerkinElmer, Waltham, MA, USA). The radical inhibition percentage was calculated by applying the equation:

RSA = [(Abs blank − Abs sample)/Abs blank] × 100

Measurements were taken in triplicate, using 12 concentrations, and the results were averaged. Triplicates represent repeated analytical measurements of the same extract and therefore reflect analytical repeatability rather than variability among independent extraction experiments. Ellagic acid was used as a positive control.

#### IC_50_ Determination

Data were statistically processed using GraphPad Prism software (Version 7.0, San Diego, CA, USA). The half-maximal inhibitory concentration (IC_50_) values were determined by fitting the concentration–response data, converted to a logarithmic scale, to a nonlinear regression model. Measurements were taken in triplicate, using 12 concentrations, and the results were averaged. Experimental data were expressed as the percentage of inhibition. Ellagic acid was used as a control, and IC_50_ values were calculated from the resulting dose–response (inhibition) curves.

### 2.6. Analysis of Ellagic Acid Content by HPLC UV/PDA

HPLC-UV/Photodiode Array (PDA) analyses were performed by an Alliance e2695 Separations Module (Waters Corporation, Milford, MA, USA) with a 20 μL loop, coupled to a Waters 2998 (PDA) detector. Data collection and processing were performed using Waters Empower 3 software (Waters Corporation, Milford, MA, USA).

A ZORBAX Eclipse XDB-C18 reversed-phase column (Agilent Technologies, Santa Clara, CA, USA), 250 mm long, with an internal diameter of 4.6 mm and a stationary phase size of 5 μm, was used to perform the chromatographic separation. Eluent A was composed of water and formic acid (99:1, *v*/*v*), while eluent B was composed of acetonitrile and formic acid (99:1, *v*/*v*) at a flow rate of 0.7 mL/min. The elution program of the solvent (A) used was as follows: 0–5 min, 90%; 5–35 min, linear decrease to 55%; 35–35.1 min, return to 90%; 35.1–40 min, equilibration at 90%. Detection was carried out with the PDA detector at 251 nm, and UV spectra were recorded between 200 and 800 nm for peak purity assessment. To ensure accurate quantification across highly divergent concentrations, a dual-range calibration strategy was adopted within the same analytical sequence using standard solutions of ellagic acid (cod. F387721, Fluorochem, UK) in methanol. A low-range calibration curve (5 points, 0–56.7 µg/mL, including the origin) was employed for the conventional chemical extract, yielding an R^2^ = 0.999. Concurrently, a high-range calibration curve (5 points, 14.2–227.0 µg/mL) was applied for the fermented samples, yielding an R^2^ = 0.997. The respective calibration equations and plots are provided in Figure S1. All samples extracted from pomegranate peel were analyzed in triplicate after filtration through a 0.45 µm membrane filter prior to injection.

### 2.7. UHPLC-DAD-HR-Orbitrap/ESI-MS Analyses of Pomegranate Peel Yeast and Chemical Extracts

The pomegranate peel yeast and chemical extracts were characterized for their chemical content by an UHPLC (Vanquish Flex) coupled with a diode array detector (DAD) and a high-resolution mass spectrometer, Q Exactive Plus Orbitrap-based FT-MS (HR-Orbitrap/MS) equipped with an electrospray ionization (ESI) source (Thermo Fisher Scientific Inc., Bremen, Germany). The dry extracts were dissolved in a methanol-water 1:1 *v*/*v* solution (UHPLC-grade from Deltek, Pozzuoli, Italy) at a concentration of 2.0 mg/mL, then centrifuged for 10 min at 4000 rpm and filtered by Phenex™ Teflon^®^ (PTFE) filter membranes (0.45 µm pore size, 47 mm diameter). Three µL of supernatant of the sample were injected into an LC-MS system using a C18 Kinetex^®^ Biphenyl column (100 × 2.1 mm, 2.6 µm particle size) provided by a security Guard^TM^ Ultra Cartridge (Phenomenex, Bologna, Italy) maintained at a temperature of 30 °C. The elution was performed at a flow rate of 0.5 mL/min with a splitting system 1:1 to DAD and MS detectors, using as a mobile phase a mixture of 0.1% *v*/*v* formic acid in water (solvent A) and 0.1% *v*/*v* formic acid in methanol (solvent B) with a linear gradient increasing from 5 to 80% solvent B over 20 min. ESI-HR mass spectra were recorded in negative ESI mode using a scan range of *m*/*z* 135–2000, both in full (70,000 resolution, 220 ms maximum injection time) and data dependent-MS/MS scan (17,500 resolution, 60 ms maximum injection time), using ionization parameters as previously reported [27]. UV spectra were recorded in the range of 200–600 nm, selecting as preferential channels 254, 280, and 325 nm. Xcalibur 4.1 software (Thermo Fisher Scientific Inc., Bremen, Germany) was used for data elaboration.

### 2.8. Antibacterial Activity Assessment

For the antibacterial investigation, both the CE and YE were tested. Ellagic acid was also included as a positive control. Each extract was tested on three different aliquots. Each aliquot was tested in triplicate.

#### 2.8.1. Bacterial Strains

A panel of six American Type Culture Collection (ATCC) strains was selected to evaluate the antimicrobial activity of the extracts. Given the preliminary nature of this study, we considered it appropriate to initially assess the activity using collection strains rather than field isolates. Among the major foodborne microorganisms of concern are *Staphylococcus aureus*, *Enterococcus faecalis*, *Listeria monocytogenes, Escherichia coli*, *Pseudomonas aeruginosa*, and *Salmonella enterica* serovar Typhi-murium. These microorganisms include important pathogenic or potentially pathogenic agents and may also contribute to the spoilage of foods of animal origin [28,29,30]. The panel comprised three Gram-positive bacteria: *Staphylococcus aureus* ATCC 6538, *Enterococcus faecalis* VANB V583E, and *Listeria monocytogenes* ATCC 7644. It also included three Gram-negative bacteria: *Pseudomonas aeruginosa* ATCC 27853, *Escherichia coli* ATCC 15325, and *Salmonella enterica* serovar Typhimurium ATCC 14028.

#### 2.8.2. Minimum Inhibitory Concentration (MIC) Determination

The antibacterial activity of the extracts was evaluated using the broth microdilution method in a 96-well polypropylene microtiter plate. Two-fold serial dilutions of the extracts were prepared, and each well of a single row was assigned to a specific bacterial strain. The MIC test was set up as follows: 95 µL of Brain Heart Infusion (BHI) broth was dispensed into each well of a 96-well polypropylene microtiter plate, except for the last three wells in each row. Each extract was reconstituted in BHI broth supplemented with 10% (*v*/*v*) DMSO to a final concentration of 1200 µg/mL. Two-fold serial dilutions were prepared in the plate from 1:2 to 1:128, resulting in final extract concentrations of 600, 300, 150, 75, 37.5, 18.75, and 9.375 µg/mL, respectively. Bacterial cultures were adjusted to a turbidity of 0.5 McFarland standard (approximately 1.5 × 10^8^ CFU/mL) and 5 µL of each culture was added to each well in the corresponding strain row, bringing the final volume in each well to 100 µL. Positive and negative controls were included for each strain in the last two wells (8th and 9th) of each row. The positive control contained only BHI broth and bacterial inoculum, while the negative control contained only BHI broth. Microplates were incubated at 37 ± 1 °C for 24 h in a humidified chamber (Heratherm IMH 100 incubator, Thermo Fisher Scientific, Waltham, MA, USA).

The MIC was determined as the lowest concentration of the extract that resulted in an approximately 80% reduction in visible bacterial growth compared to the positive control [31]. Each extract-strain combination was tested in triplicate, and the MIC value was determined as the mode of the replicate values. Results were expressed as μg/mL of the extract.

#### 2.8.3. Determination of Minimum Bactericidal Concentration (MBC)

To determine the minimum bactericidal concentration, a drop of each sample was plated onto Tryptic Soy Agar (TSA) plates (Thermo Fisher Scientific S.p.A., Segrate, Milan, Italy) and incubated aerobically at 37 ± 1 °C for 24 h. Starting from the well corresponding to the MIC value and moving towards higher concentrations, a drop of each sample was inoculated into each well. The MBC is the lowest concentration of the sample that showed no visible bacterial growth. Results were expressed as μg/mL of the extract.

### 2.9. Statistical Analysis

Data were statistically processed using JMP 7 software (SAS Institute, 2008). For the statistical analysis of MIC and MBC values, the value “> 600 µg/mL” was taken as 1200 µg/mL because this would have been twice the maximum concentration tested. The microbiological analysis was processed as follows. After checking that the data were not normally distributed using the Shapiro–Wilk test and testing for homogeneity of variances using the Bartlett test. Differences among treatments were assessed by using the non-parametric Kruskal–Wallis H-test, followed by post hoc Mann–Whitney U-test pairwise comparisons. *p*-values were corrected using the Benjamini–Hochberg procedure. Differences with *p* < 0.05 were considered statistically significant.

## 3. Results

### 3.1. DPPH Radical Scavenging Capacity

**Figure 1:**
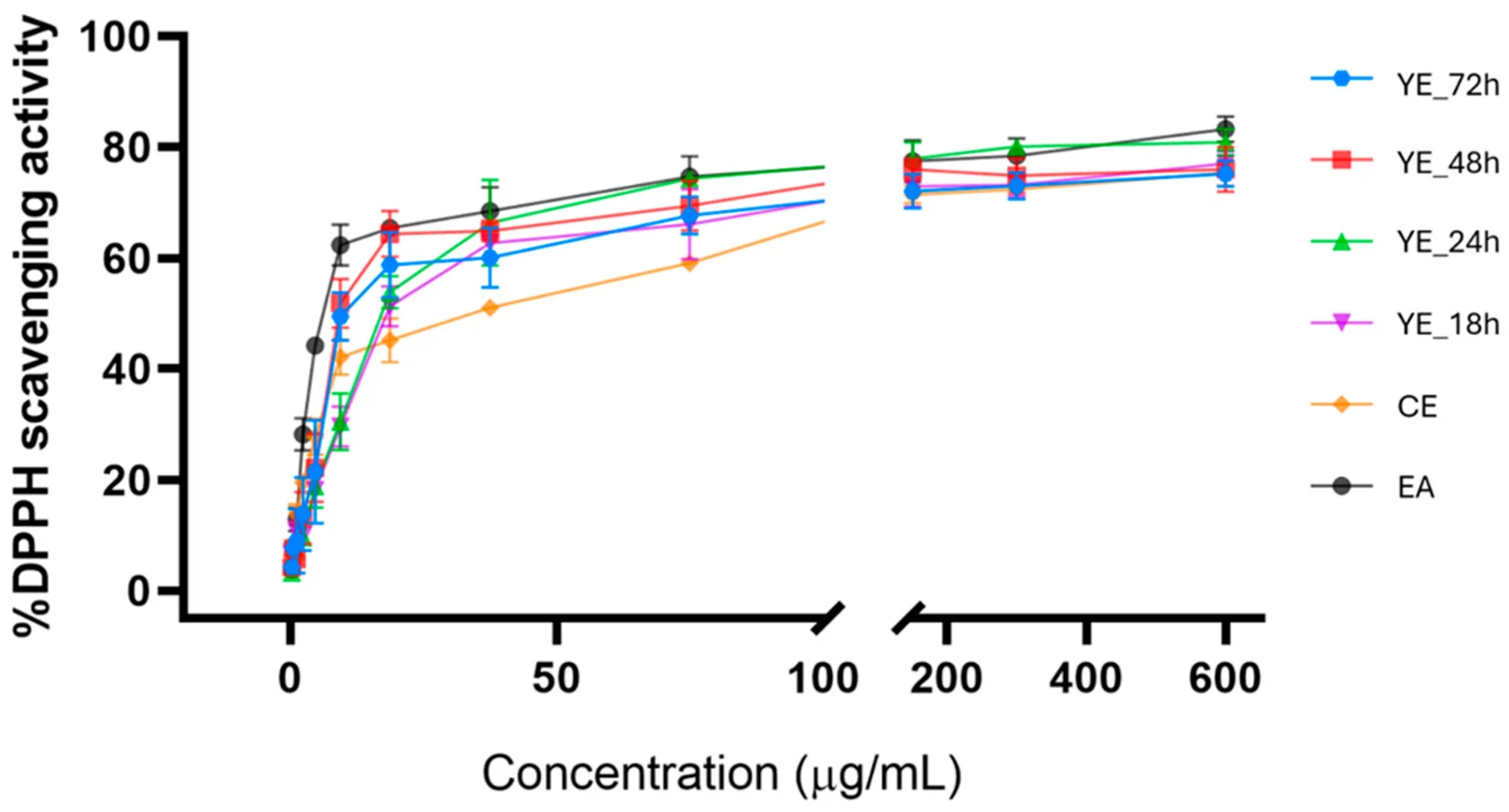
Scavenging activity (%) of pomegranate peel extracts: Chemical Extract (CE) and Yeast Extract (YE) after 18, 24, 48 and 72 h of fermentation. Ellagic Acid (EA) was used as a reference. Data are expressed as mean ± SD of triplicate measurements; error bars represent the standard deviation.

The curves of different scavenging activities of both CE and YE pomegranate peel extracts were reported in Figure 1. The calculated antioxidant capacity (IC_50_ values), reported in Table 1, revealed a marked enhancement of antioxidant activity following yeast fermentation, with IC_50_ ranging from 15.11 to 8.57 μg/mL. While the chemical pomegranate extract (CE) exhibited the weakest radical scavenging activity across the entire concentration range tested (1.17–600 μg/mL), fermentation markedly improved the antioxidant potential of the peel extracts. This effect was evidenced by a time-dependent decrease in IC_50_ values, indicating a progressive increase in scavenging efficiency with fermentation duration. The greatest potency was observed after 48 h of fermentation, with the corresponding extract displaying an IC_50_ value of 8.57 μg/mL, approximately five-fold lower than that of the chemical extract (43.6 μg/mL), even though ellagic acid, used as the reference antioxidant, exhibited the strongest activity, with the lowest IC_50_ value (1.57 μg/mL). Overall, the results demonstrate that yeast fermentation is an effective strategy for enhancing the radical scavenging capacity of pomegranate peel extracts compared with conventional extraction methods.

### 3.2. Analysis of Ellagic Acid Content

**Figure 2:**
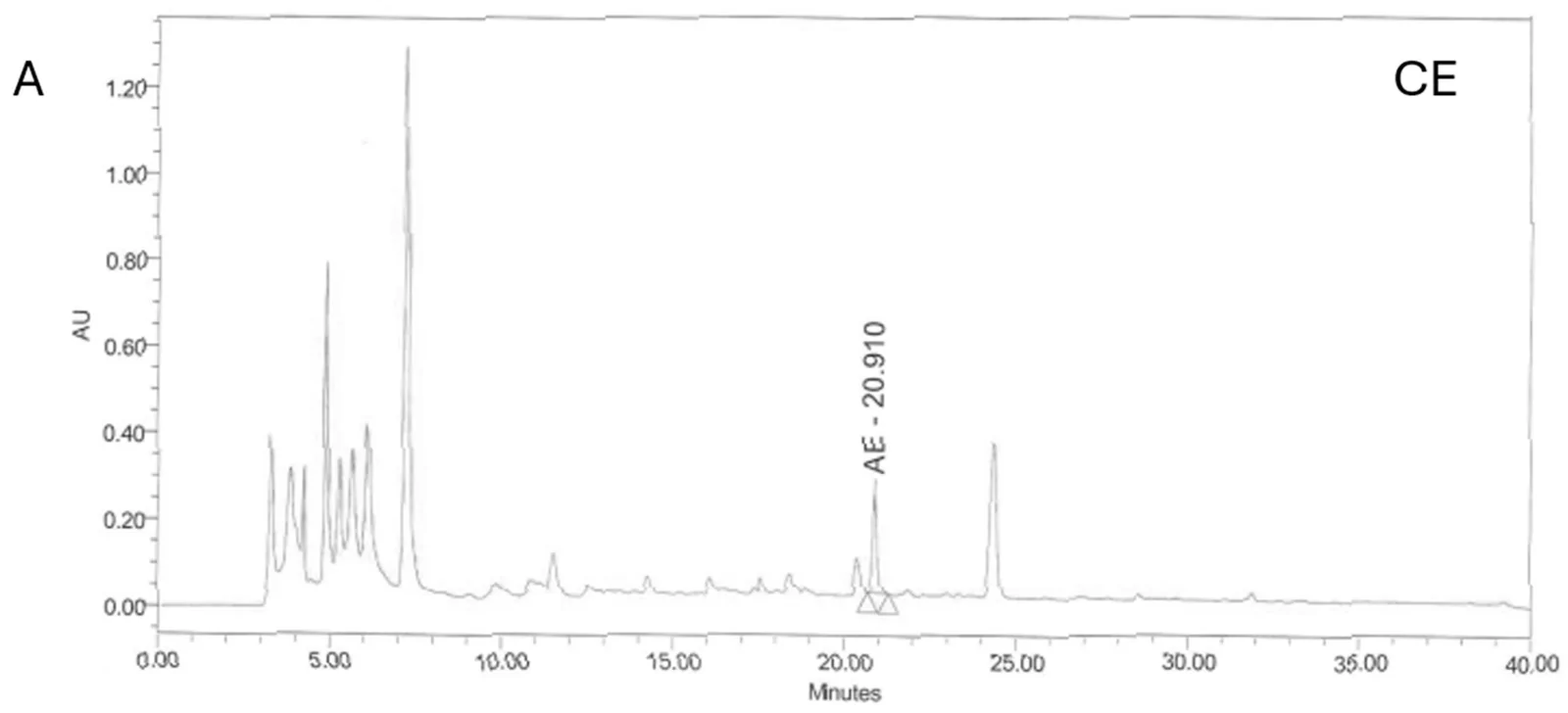

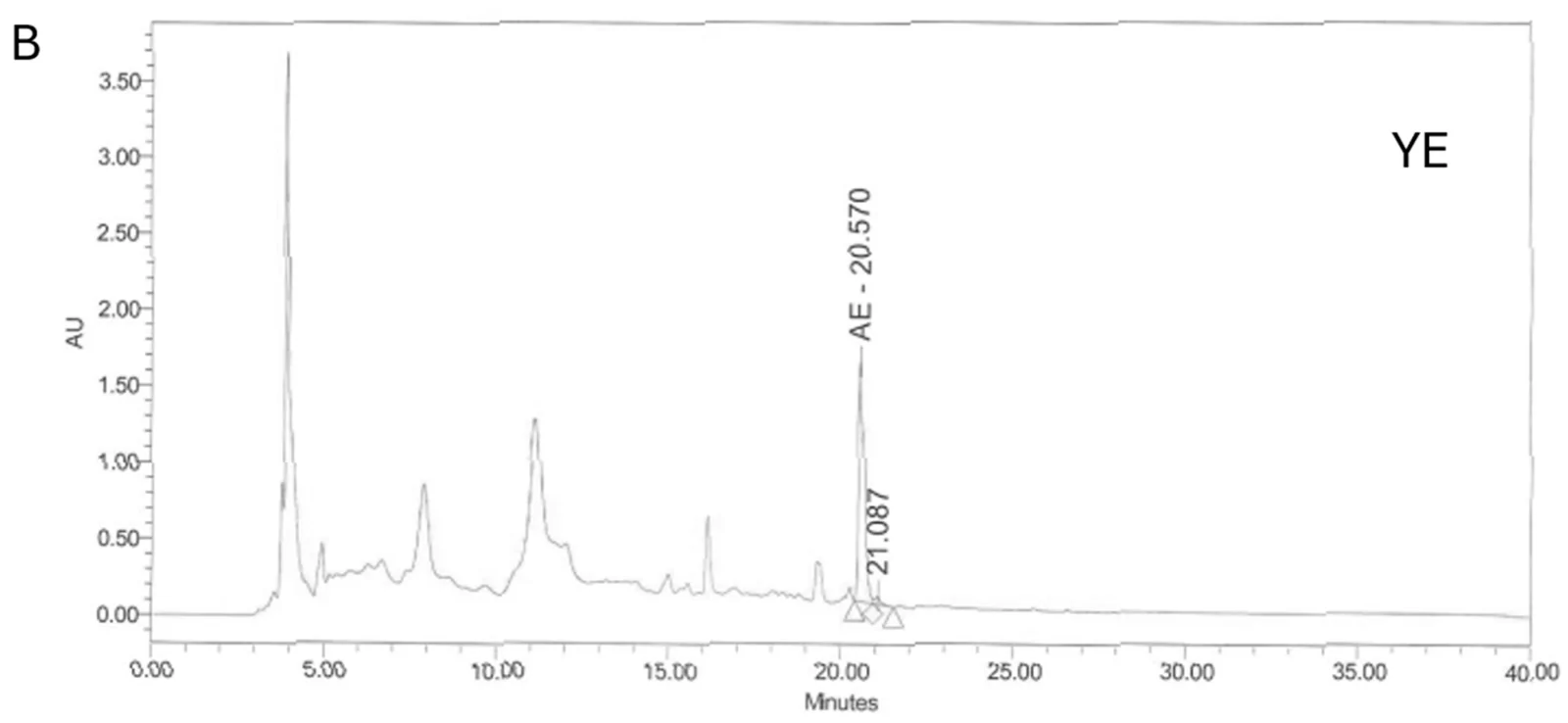
Representative HPLC–PDA chromatograms of pomegranate peel extracts showing the Ellagic Acid (EA) peak at approximately 20.7 min. (**A**) CE: extract obtained through chemical extraction; (**B**) YE_48h: extract obtained after 48 h of yeast-mediated extraction. Chromatographic conditions: ZORBAX Eclipse XDB-C18 column (250 × 4.6 mm, 5 μm); mobile phase: water (0.1% formic acid) and acetonitrile (0.1% formic acid); flow rate: 0.7 mL/min; detection at 251 nm. The Y-axis represents relative absorbance (%), normalized to the highest peak of each chromatogram.

Given the highest antioxidant activity exhibited by YE_48h, a quantitative analysis of its ellagic acid content was performed using HPLC-UV-DAD and compared to that present in CE. Among the phenolic compounds of pomegranate peel, ellagic acid was selected as the target analyte of this determination, as it is the main hydrolysis product of ellagitannins and its release is directly promoted by the fermentation process [24]; the remaining bioactive constituents were characterized qualitatively by UHPLC-HRMS, as reported in Section 3.3. Chromatographic peaks corresponding to ellagic acid were identified in both chemical (CE) and yeast extracts (YE) of pomegranate peel by comparing their retention times (Figure 2) and UV–Vis spectral profiles with those of the reference standard (Figures S2 and S3). The ellagic acid content of CE and YE_48h was 3.3 μg/mL and 59.3 μg/mL, respectively. YE_48h exhibited an approximately 18-fold higher concentration of ellagic acid than CE. This marked increase indicates that the enzymatic and metabolic activity of *S. cerevisiae* during fermentation enhanced the release of ellagic acid from the peel matrix.

### 3.3. Chemical Fingerprint of Pomegranate Peel Yeast Extract and Chemical Extracts

**Figure 3:**
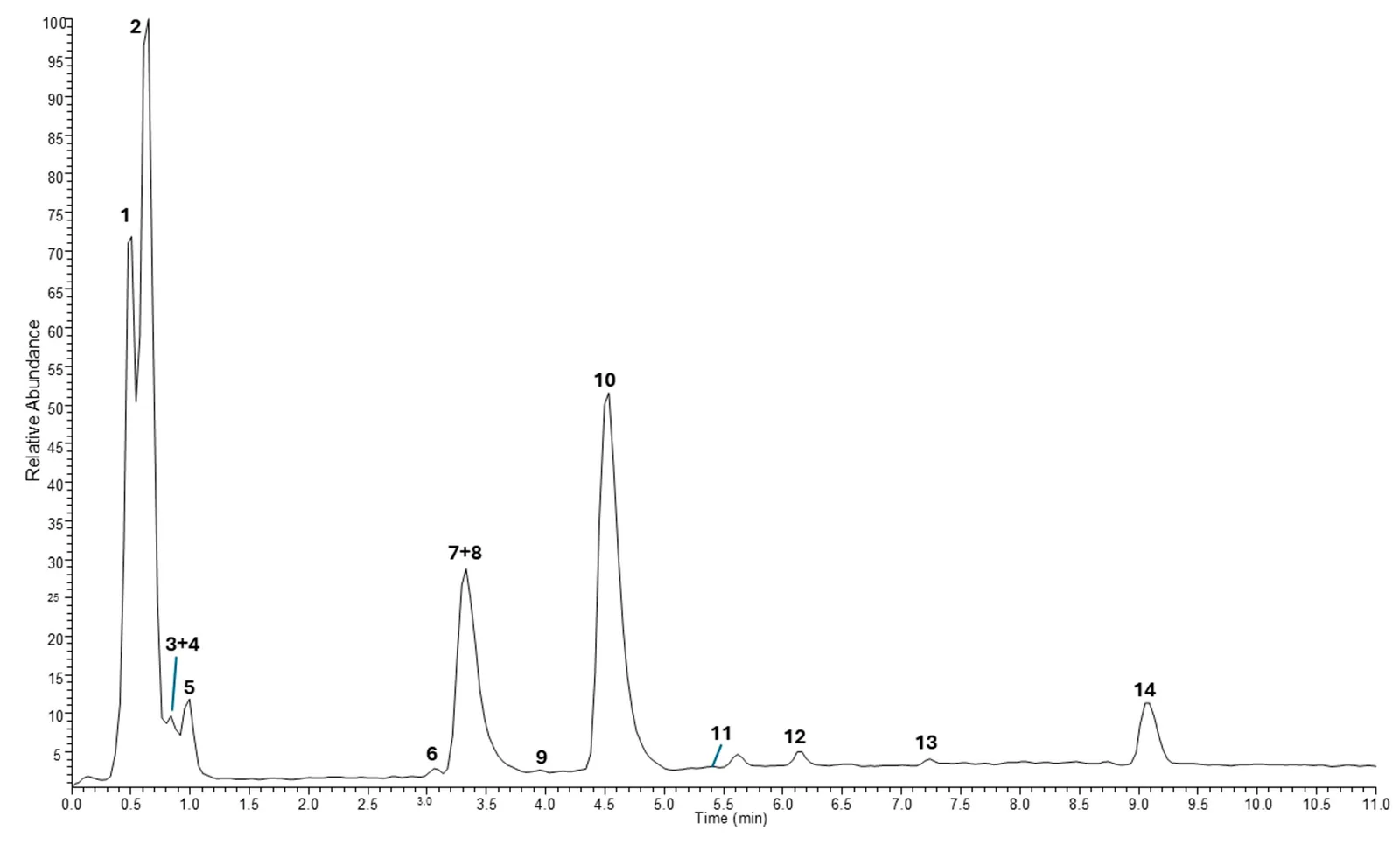
LC/Orbitrap/ESI-MS profile of pomegranate peel extract YE_48h obtained by treating with yeast for 48 h. Data are expressed as relative absorbance (Y-axis) as a function of retention time (min, X-axis). Peak data are reported in Table 2.

The chemical fingerprint of both the yeast and chemical extracts was investigated by UHPLC-HR-MS analyses (Figure 3 and Figure S4, respectively). Results showed that both extracts shared few components, while the chemical profile of YE_48h extract was characterized by the presence of further specialized metabolites, such as ellagitannins, typically found as constituents of pomegranate peels.

All compounds were tentatively identified based on comparison of their molecular formula and both ESI-MS and ESI-MS/MS data with the literature data [32,33,34], with an accepted mass error < 5 ppm. The identified components include gallic acid (5) and ellagic acid (14), together with their derivatives. According to the literature [34], the most represented compound is punicalagin, occurring in two different isomeric forms (α and β anomers, compounds 7 and 10), detected as a doubly charged molecular deprotonated ion ([M − 2H]^2−^) at *m*/*z* 541.0263. Other ellagitannins occurred as bis-hexahydroxydiphenoyl hexoside (HDPH) derivatives such as pedunculagin in two isomeric forms (8 and 9), galloyl-HHDP-hexoside (11), digalloyl-HHDP-hexoside (12), and di-(HHDP-galloyl-hexoside)-pentoside (6), as deduced by the presence in MS/MS experiments of the product ion at *m*/*z* 301.00 corresponding to deprotonated ellagic acid. Hexosides of gallic acid (3), HHDP (4), and ellagic acid (13) were also detected (Table 2 and Table S1).

### 3.4. Antibacterial Activity

To confirm that the fermentation of food by-products can represent a valuable strategy for the recovery of bioactive compounds, we assessed the antibacterial activity of the YE_48h extract and compared it with that of the CE extract, using ellagic acid as a positive control.

MIC and MBC values were reported in Table 3 and Table S2, and Table 4 and Table S3, respectively. Ellagic acid had MIC values lower than those of both experimental extracts CE and YE_48h on *S. aureus* (*p* = 0.0379 for both extracts), *L. monocytogenes* (*p* = 0.0253 for both extracts) and *S. typhimurium* (*p* = 0.0379 for both extracts). The CE peel pomegranate extract had MIC values lower than YE_48h on *S. aureus*, *L. monocytogenes* and *E. faecalis* (*p* = 0.0379). As concerns *E. faecalis*, CE had the lowest MIC value.

The bactericidal activity did not differ significantly among the extracts evaluated, irrespective of the bacterial strain tested (Table 4). With regard to *P. aeruginosa*, there was a trend bordering on non-significance, in which the extract prepared using yeast showed greater activity than the chemical extract, comparable to that of ellagic acid (*p* = 0.05085).

## 4. Discussion

In line with circular economy principles, this study aimed to develop an environmentally sustainable strategy for the valorization of pomegranate peel waste through the recovery of bioactive compounds without the use of organic solvents, chemical reagents, or elevated temperatures. For this purpose, an eco-friendly extraction process based on the fermentative activity of *S. cerevisiae* was investigated at different incubation times (18, 24, 48, and 72 h). This approach is in line with the growing body of literature recognizing fermentation as a key biotechnology of the circular bioeconomy for the valorization of agri-food wastes and the recovery of high-value bioproducts [35]. The resulting extracts were evaluated for their antioxidant activity, while the chemical profile, the ellagic acid content and the antibacterial properties were assessed for the extract showing the most promising biological activity.

The antioxidant activity of the fermented extracts was assessed by determining their IC_50_ values. Among all samples evaluated, the YE_48h extract exhibited the greatest antioxidant capacity, demonstrating superior efficacy relative to the extracts obtained at different fermentation times as well as to the chemically extracted sample (CE). Nevertheless, both YE_48h and CE displayed lower antioxidant potency than pure ellagic acid, used as the reference compound. Extending the fermentation process to 72 h resulted in a slight reduction in antioxidant activity. This decrease may be related to the inhibitory effects exerted by pomegranate-derived phytochemicals on yeast metabolism. As fermentation proceeds, the concentration of ellagic acid, the only phenolic compound quantified in this study, increases, potentially affecting yeast viability and reducing the extraction efficiency, as previously reported for pomegranate-derived bioactive constituents [36,37]. It should be noted that, apart from ellagic acid, the phenolic profile was assessed only qualitatively; therefore, no quantitative conclusions on the changes in concentration of the other phenolic constituents can be drawn.

Based on its greater antioxidant activity among the fermented samples, YE_48h was then selected for further phytochemical investigation. Quantitative HPLC analysis showed a remarkable enhancement in ellagic acid content, with YE_48h containing approximately 18-fold higher levels of ellagic acid than the chemical extract. This finding suggests that the fermentation process, conducted at controlled time, temperature and pH [24], effectively promotes the release of ellagic acid from ellagitannin-rich pomegranate peel waste. It can be hypothesized that the enzymatic and metabolic activities associated with *S. cerevisiae* may facilitate the hydrolysis of complex polyphenolic structures, increasing the availability of free ellagic acid and enhancing the functional activity of the extract. This process could contribute to the valorization of pomegranate peel waste through a sustainable and low-energy process [38]. However, in the absence of direct measurements of enzymatic activities and fermentation-derived metabolites, these mechanistic interpretations remain hypothetical and require dedicated experimental validation.

To further elucidate the phytochemical composition of YE_48h, UHPLC-HRMS fingerprint analysis was performed. Compared to CE (Figure S4), YE_48h (Figure 3) showed, in addition to the significant increase in ellagic acid content, additional water-soluble bioactive phenolics, including punicalagin isomers I and II and pedunculagin isomers I and II; only gallic acid and ethyl gallate occurred in both samples. These hydrolysable tannins are well recognized for their antioxidant and antimicrobial properties and have been identified as major contributors to the biological activity of pomegranate-derived products [39]. In particular, pedunculagin has recently been reported to exert antibacterial activity against *S. aureus*, supporting a potential role of this compound in the antimicrobial effects [40].

The antibacterial activity of pomegranate peel extracts was evaluated against both Gram-positive and Gram-negative strains. Among the Gram-positive species, *S. aureus* and *E. faecalis* are common human-associated bacteria that colonize the skin, mucosal surfaces, and gastrointestinal tract, whereas *L. monocytogenes* is principally an environmental and foodborne pathogen. Among the Gram-negative species, *P. aeruginosa* is ubiquitous in soil and aquatic environments, while *E. coli* and *S. typhimurium* are primarily associated with the intestinal tract of humans and animals. The obtained results indicate that fermentation with *S. cerevisiae* can modulate the antibacterial activity of pomegranate peel extracts against Gram-positive bacteria in a strain-dependent manner. Notably, improved activity was observed against *S. aureus*, suggesting that fermentation may increase the availability of existing antimicrobial compounds or promote the formation of new bioactive metabolites through biotransformation processes [41]. However, targeted profiling of fermentation-derived metabolites is required to confirm this hypothesis.

Conversely, no enhancement of activity was detected against *L. monocytogenes*, while both *E. coli* and *S. typhimurium* remained resistant to the extracts. These findings indicate that the antimicrobial activity of the fermented extract cannot be explained exclusively by the increased concentration of ellagic acid. Rather, the overall antimicrobial profile likely results from the combined action of multiple phenolic constituents and fermentation-derived metabolites. We hypothesize that synergistic interactions among ellagic acid, punicalagins, pedunculagin, and other minor compounds may contribute to the selective antibacterial effects observed, highlighting the complexity of the phytochemical matrix generated during fermentation. Furthermore, many compounds that do not exhibit antioxidant activity may nevertheless possess antibacterial properties, including volatile fatty acids and other carboxylic acids. Further characterization of the extract could therefore provide a more comprehensive understanding of its chemical composition and identify additional compounds with antibacterial activity, which may act independently or synergistically with those already identified [42,43].

Overall, both the fermented and chemically extracted samples exhibited greater efficacy against Gram-positive than Gram-negative bacteria. Similar observations have been previously reported for pomegranate-derived extracts and other polyphenol-rich plant products [44]. This differential susceptibility is generally attributed to differences in bacterial cell wall structures. The outer membrane of Gram-negative bacteria acts as an additional permeability barrier that limits the penetration of many antimicrobial compounds, whereas Gram-positive bacteria lack this protective layer, resulting in greater susceptibility to the effects of polyphenols.

Among the Gram-positive strains tested, the highest bactericidal activity was observed against *S. aureus* and *E. faecalis*, both of which represent important foodborne and opportunistic pathogens [28]. *S. aureus* is a major cause of food contamination and food poisoning due to its ability to produce heat-stable enterotoxins in dairy products, meat products, and other processed foods. Likewise, *E. faecalis* is widely distributed in food and environmental sources and is increasingly recognized as an opportunistic pathogen associated with severe human infections and antimicrobial resistance [28,29]. The effectiveness of the fermented extract against these clinically and food-relevant microorganisms further supports the potential application of yeast-assisted pomegranate peel valorization as a sustainable strategy for the production of natural antimicrobial agents.

## 5. Conclusions

This study demonstrates that fermentation through *S. cerevisiae* is an effective, sustainable, and solvent-free approach for the valorization of pomegranate peel, an agro-industrial by-product of increasing interest in the health products field. The fermentation process significantly enhanced the release of ellagic acid, yielding extracts with antioxidant activity greater than that obtained by conventional chemical extraction. Moreover, the extract obtained after 48 h of fermentation (YE_48h) proved to modulate the antibacterial activity against selected pathogenic bacteria. Overall, these findings highlight the potential of yeast-assisted fermentation as a green technology for recovering high-value bioactive compounds from food processing waste and for generating functional extracts with antioxidant and antimicrobial properties. The combination of sustainable extraction of pomegranate peel with a comprehensive analytical and functional characterization represents a promising strategy for the valorization of agro-industrial waste in line with circular economy principles. Nevertheless, further biological and phytochemical investigations are required to fully characterize the fermented extract, identify the compounds responsible for its bioactivity, and assess its safety and efficacy in relevant application models. Altogether, these studies will contribute to expanding its potential use in the food, nutraceutical, and pharmaceutical sectors.

## Figures and Tables

**Table 1:** IC_50_ values of pomegranate peel extracts: CE and YE after 18, 24, 48 and 72 h. Ellagic Acid (EA) was used as a reference.

	IC_50_ (μg/mL)
EA	1.57
CE	43.62
YE_72h	9.8
YE_48h	8.57
YE_24h	13.40
YE_18h	15.11

**Table 2:** Chromatographic and negative ion mode HR-ESI-MS/MS data of constituents tentatively identified in pomegranate peel extract YE_48h obtained by treating with yeast for 48 h.

Peak ^a^	Compound	*t*_R_ (min)	[M − H]^−^ (*m*/*z*)	ESI-MS/MS Product Ions ^b^ (*m*/*z*)	Molecular Formula	Error (ppm)
**1**	Hexose	0.5	179.0559	161.05, 89.02, 71.01, **59.01**	C_6_H_12_O_6_	−1.117
**2**	Citric acid	0.7	191.0193	173.00, **111.01**, 87.00	C_6_H_8_O_7_	−2.251
**3**	Galloyl hexoside	0.8	331.0674	271.05, 211.02, 169.01, 151.00	C_13_H_16_O_10_	+0.906
**4**	HHDP-hexoside	0.8	481.0625	301.00, 275.02	C_20_H_18_O_14_	−0.249
**5**	Gallic acid	1.0	169.0137	**125.02**, 97.03, 79.02, 69.03	C_7_H_6_O_5_	+3.254
**6**	Di-(HHDP-galloyl-hexoside)- pentoside	3.1	707.0642 ([M − 2H]^2−^)	783.07, 633.07, 613.05, 481.06, **301.00**	-	-
**7**	Punicalagin isomer I (HHDP-gallagyl-glucoside)	3.3	541.0263^c^ ([M − 2H]^2−^)	781.05, 600.99, 575.01, 301.00, 275.02	C_48_H_28_O_30_	−0.573
**8**	Pedunculagin isomer I (bis-HHDP-glucoside)	3.3	783.0694	481.06, **301.00**, 275.02	C_34_H_24_O_22_	−0.958
**9**	Pedunculagin isomer II (bis-HHDP-glucoside)	4.0	783.0694	481.06, **301.00**, 275.02	C_34_H_24_O_22_	−0.958
**10**	Punicalagin isomer II (HHDP-gallagyl-glucoside)	4.5	541.0263 ([M − 2H]^2−^)	781.05, 600.99, 575.01, 301.00, 275.02	C_48_H_28_O_30_	−0.573
**11**	Galloyl-HHDP-hexoside	5.4	633.0735	463.05, **301.00**, 275.02	C_27_H_22_O_18_	+0.316
**12**	Digalloyl-HHDP-hexoside	6.2	785.0857	483.07, 419.06, **301.00**, 275.02	C_34_H_26_O_22_	+1.783
**13**	Ellagic acid hexoside	7.2	463.0523	**301.00**,	C_20_H_16_O_13_	+1.080
**14**	Ellagic acid	9.1	300.9991	257.01, 245.01, 145.03	C_14_H_6_O_6_	+0.332

^a^ Compounds are listed in elution order, and peak numbers correspond with those of Figure 3. ^b^ The base ion peak is shown in bold. HHDP = bis-hexahydroxydiphenoyl hexoside; *t*_R_ = retention time.

**Table 3:** MIC values (expressed as mode) of two kinds of pomegranate peel extracts: Chemical Extract (CE) and extract after 48 h of a yeast treatment (YE_48h). The activity of Ellagic Acid (EA) is evaluated as a positive control. Different letters written in superscript on the same row indicate significant differences at the Kruskal–Wallis H-test, followed by post hoc Mann–Whitney U-test pairwise comparisons and corrected using the Benjamini–Hochberg procedure, for *p* < 0.05. The reported *p*-value corresponds to the overall Kruskal–Wallis test performed across all three groups.

Bacterial Strains	MIC (μg/mL)			
	EA	CE	YE_48h	*p* Value
*Staphylococcus aureus*	37.5 ^b^	75 ^a^	75 ^a^	0.0183
*Listeria monocytogenes*	75^c^	150^b^	600^a^	0.0183
*Enterococcus faecalis*	300^a^	150^b^	300^a^	0.0183
*Escherichia coli*	300	300	300	1
*Pseudomonas aeruginosa*	300	300	300	1
*Salmonella enterica* serovar Typhimurium	150^b^	300^a^	300^a^	0.0183

**Table 4:** MBC values (expressed as mode) of two kinds of pomegranate peel extracts: Chemical Extract (CE) and extract after 48 h (YE_48h) of a yeast treatment. The activity of EA is evaluated as a positive control. The reported *p*-value corresponds to the overall Kruskal–Wallis test performed across all three groups.

Bacterial Strains	MBC (μg/mL)			
	EA	CE	YE_48h	*p* Value
*Staphylococcus aureus*	150	75	150	0.2636
*Listeria monocytogenes*	150	600	>600	0.0319
*Enterococcus faecalis*	600	600	300	0.6703
*Escherichia coli*	>600	>600	>600	1
*Pseudomonas aeruginosa*	300	>600	600	0.0289
*Salmonella enterica* serovar Typhimurium	>600	>600	>600	1

## Data Availability

The original contributions presented in this study are included in the article/Supplementary Materials. Further inquiries can be directed to the corresponding author.

## Supplementary Materials

The following supporting information can be downloaded at https://www.mdpi.com/article/10.3390/foods15203764/s1. Figure S1. HPLC calibration curve of ellagic acid.; Figure S2. UV-Vis absorption spectrum of EA; Figure S3. HPLC chromatogram and UV-Vis spectrum of the YE_48h extract. Figure S4. LC/Orbitrap/ESI-MS profile of pomegranate chemical extract (CE). Table S1. Chromatographic and negative ion mode HR-ESI-MS/MS data. Table S2. Significant differences among treatments for each bacterial strain for MIC values; Table S3. Significant differences among treatments for each bacterial strain for MBC values.
