# Supplementary material for: Development of Sustainable Extraction Processes for the Recovery of Antioxidants from Punica granatum L. Peel
Source: Foods. 2026 Oct 21;15(20):3764. doi: 10.3390/foods15203764 (PMC13704383; doi:10.3390/foods15203764)
Supplement: Supplementary file 1 [file foods-15-03764-s001.zip › foods-4590413-supplementary.pdf]

Supplementary info index

Figure S1. HPLC calibration curve of ellagic acid (high-range and Low range).

Figure S2. UV-Vis absorption spectrum of ellagic acid (EA).

Figure S3. HPLC chromatogram and UV-Vis spectrum of the YE_48h extract.

Figure S4. LC/Orbitrap/ESI-MS profile of pomegranate chemical extract (CE).

Table S1. Chromatographic and negative ion mode HR-ESI-MS/MS data of constituents tentatively identified in pomegranate peel chemical extract (CE).

Table S2. Significant differences among treatments for each bacterial strain for MIC values

Table S3. Significant differences among treatments for each bacterial strain for MBC values

Calibration curve

Processing Method: EA RP_HPLC

A	1.619120e+006
B	2.884697e+005
C	0.000000e+000
D	0.000000e+000
R^2	0.997294

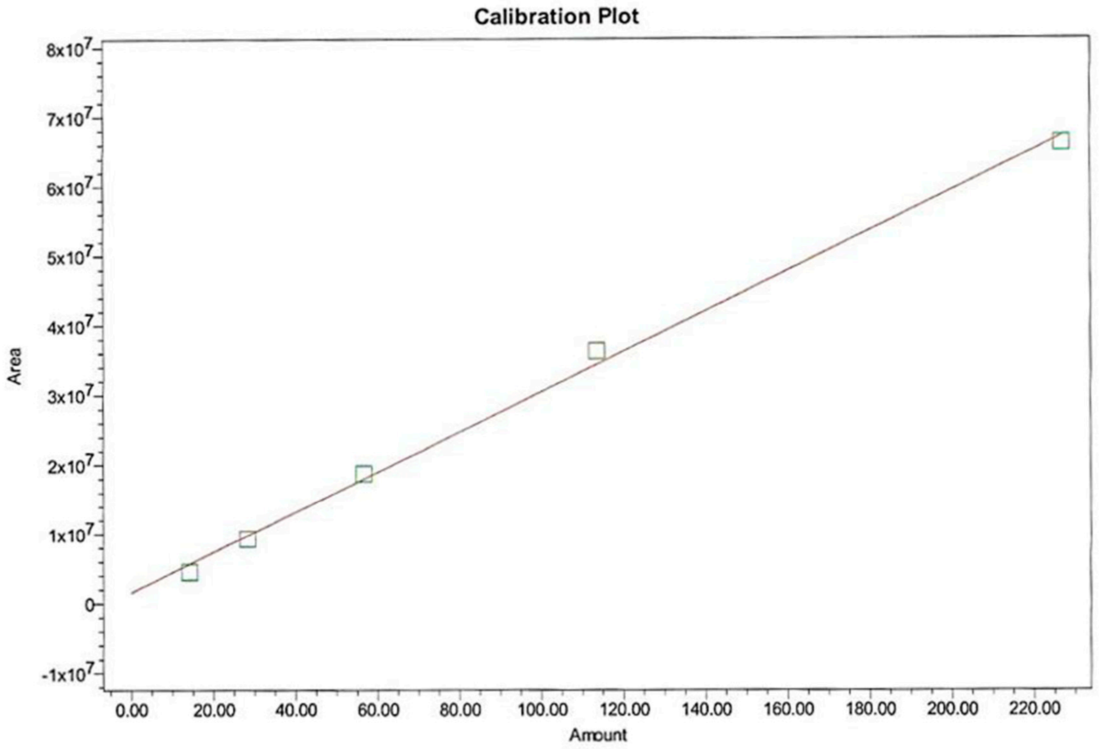

	Name	X Value	Response	Calc. Value	% Deviation	Manual	Ignore
**1**	EA	14.200000	4568020.577883	10.222567	-28.010	No	No
**2**	EA	28.370000	9326984.243058	26.719842	-5.817	No	No
**3**	EA	56.750000	18695851.317242	59.197665	4.313	No	No
**4**	EA	113.500000	36285559.662681	120.173599	5.880	No	No
**5**	EA	227.000000	66093918.299700	223.506326	-1.539	No	No

Figure S1A. High-range HPLC calibration curve of ellagic acid (EA) spanning from 14.2 to 227.0 µg/mL (R^2 = 0.9973). This curve was applied for the accurate quantification of the fermented pomegranate peel extracts (YE_48h), which exhibited high analyte concentrations.

Calibration Curve

Processing Method: EA RP_HPLC

A	5.279648e+003
B	3.288190e+005
C	0.000000e+000
D	0.000000e+000
R^2	0.999899

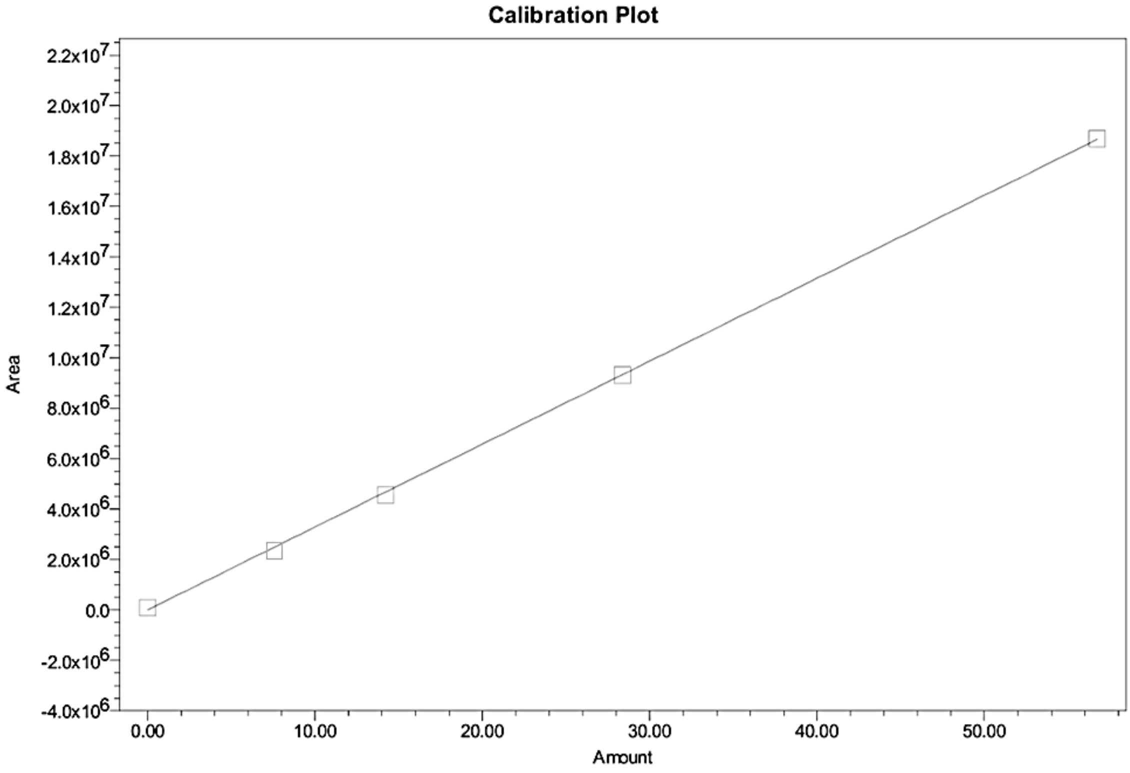

	Name	X Value	Response	Calc. Value	% Deviation	Manual	Ignore
**1**	EA	0.000000	88569.715703	0.253301		No	No
**2**	EA	7.100000	2313746.861120	7.020480	- 1.120	No	No
3	EA	14.200000	4568020.577883	13.876146	-2.281	No	No
4	EA	28.370000	9326984.243058	28.349041	-0.074	No	No
5	EA	56.750000	18695851.317242	56.841512	0.161	No	No

Figure S1B. Low-range HPLC calibration curve of ellagic acid (EA) spanning from 0.0 to 56.7 μg/mL (R^2 = 0.9999). This curve, which includes the origin (blank point), was generated within the same analytical sequence to guarantee the reliable quantification of low-concentration samples (CE).

absorption spectrum of standard EA

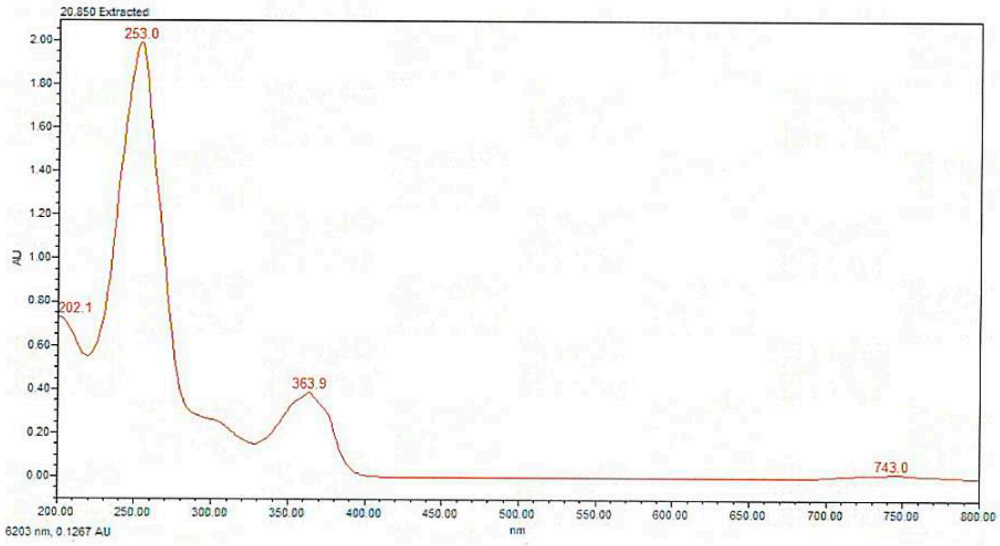

Figure S2. UV-Vis absorption spectrum of ellagic acid (EA), showing a principal absorption maximum at 253.0 nm and a secondary band at 363.9 nm, consistent with the characteristic spectral profile of ellagic acid.

A

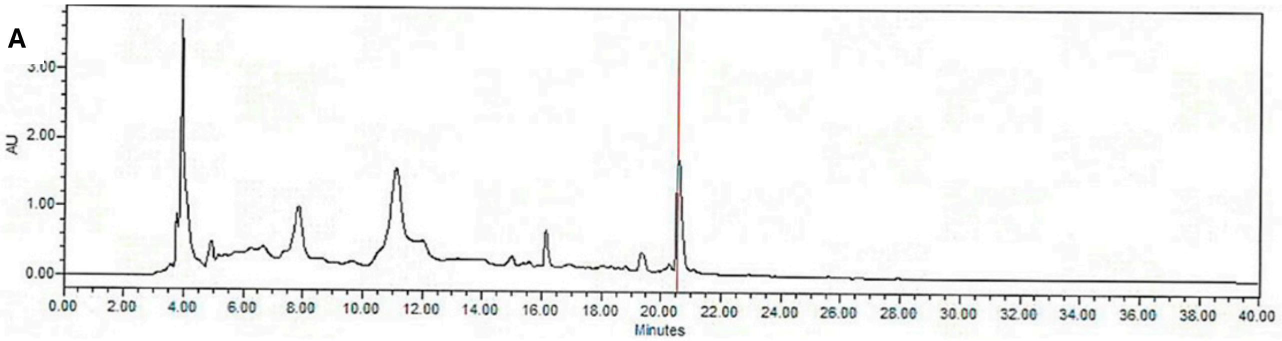

B

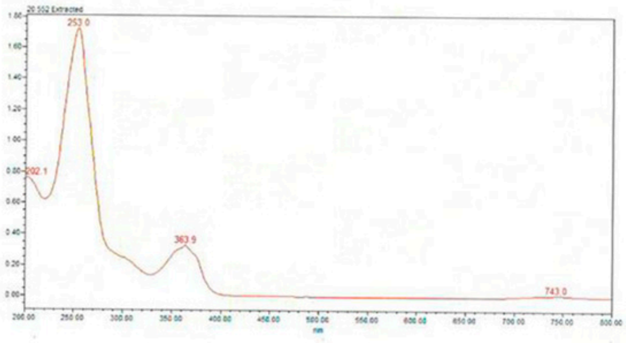

Figure S3. A. HPLC chromatogram of the YE_48h extract. The major peak detected at approximately 20.9 min was assigned to ellagic acid (EA). **B**. UV-Vis absorption spectrum of the chromatographic peak assigned to EA, showing characteristic absorption maxima at 253.0 and 363.9 nm.

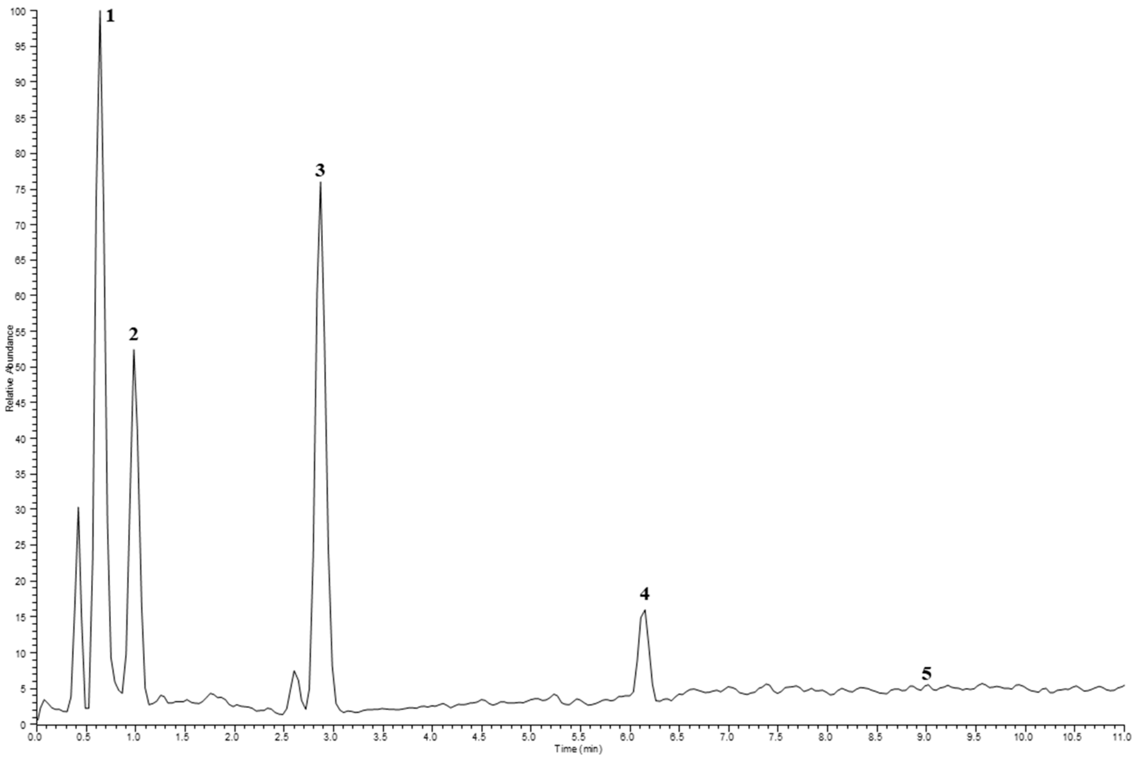

Figure S4. LC/Orbitrap/ESI-MS profile of pomegranate chemical extract (CE). Peak data are reported in Table S1.

Table S1. Chromatographic and negative ion mode HR-ESI-MS/MS data of constituents tentatively identified in pomegranate peel chemical extract (CE).

**Peak**[a]	**Compound**	t_R **(min)**	$[M-H]^-$ **(*m/z*)**	**ESI-MS/MS Product Ions**[b] **(*m/z*)**	**Molecular formula**	**Error (ppm)**
1	Citric acid	0.6	191.0193	173.00, **111.01**, 87.00	$C_6H_8O_7$	-2.251
2	Gallic acid	1.0	169.0137	**125.02**, 97.03, 79.02, 69.03	$C_7H_6O_5$	+3.254
3	Ethyl citrate	2.9	219.0508	173.01, **111.01**, 87.00	$C_8H_{12}O_7$	-0.913
4	Ethyl gallate	6.1	197.0453	169.01, 125.02, 97.03, 79.02, 69.03	$C_9H_{10}O_5$	-1.015
5	Ellagic acid	9.1	300.9991	257.01, 245.01, 145.03	$C_{14}H_6O_6$	+0.332

[a]Compounds are listed in elution order, and peak numbers correspond with those of Figure S4. [b] The base ion peak is shown in bold. t_R = retention time.

Table S2 Pairwise statistical comparison of minimum inhibitory concentration (MIC) values for ellagic acid (EA), chemical extract (CE), and yeast-fermented extract (YE) against the tested bacterial strains. Values represent *p*-values obtained from pairwise statistical comparisons of MIC values. *Statistically significant difference at $p < 0.05$. A *p*-value ≥ 0.05 indicates no statistically significant difference.

Bacterial strains	EA *vs* CE	CE *vs* YE	EA *vs* YE
	***p*-value**	***p*-value**	***p*-value**
Staphylococcus aureus	0.0379*	1	0.0379*
Listeria monocytogenes	0.0253*	0.0253*	0.0253*
Enterococcus faecalis	0.0379*	0.0379*	1
Salmonella enterica serovar Typhimurium	0.0379*	1	0.0379*

Table S3 Pairwise comparisons of MBC values for ellagic acid (EA), chemical extract (CE), and yeast-fermented extract (YE) against the tested bacterial strains. Values represent *p*-values obtained from pairwise statistical comparisons of MBC values. A *p*-value ≥ 0.05 indicates no statistically significant difference.

Bacterial strains	EA *vs* CE	CE *vs* YE	EA *vs* YE
	***p*-value**	***p*-value**	***p*-value**
Listeria monocytogenes	0.0646	0.1138	0.0646
Pseudomonas aeruginosa	0.0508	0.0508	0.1138
